# Trading Patients: Applying the Alternative Model for Personality Disorders to Two Cases of *DSM-5* Borderline Personality Disorder Over Time and Across Therapists

**DOI:** 10.3389/fpsyg.2022.794624

**Published:** 2022-02-14

**Authors:** Chloe F. Bliton, Lia K. Rosenstein, Aaron L. Pincus

**Affiliations:** Department of Psychology, The Pennsylvania State University, University Park, PA, United States

**Keywords:** Alternative Model for Personality Disorders (AMPD), borderline personality disorder (BPD), personality assessment, transference focused psychotherapy (TFP), clinical utility

## Abstract

The *DSM*-*5* Alternative Model for Personality Disorders (AMPD) dimensionally defines personality pathology using severity of dysfunction and maladaptive style. As the empirical literature on the clinical utility of the AMPD grows, there is a need to examine changes in diagnostic profiles and personality expression in treatment over time. Assessing these changes in individuals diagnosed with borderline personality disorder (BPD) is complicated by the tendency for patients to cycle through multiple therapists over the course of treatment leaving the potential for muddled diagnostic clarity and disjointed case conceptualizations. Following patient trajectories across therapists offers a unique opportunity to examine the AMPD’s sensitivity to and utility for capturing personality stability and change over time for patients with BPD. This article demonstrates the utility of the AMPD for two clinical cases in three distinct ways: (i) highlighting heterogeneity in BPD between patients, (ii) comparing improvements in personality severity and style over time, and (iii) elucidating profile change across therapist ratings. We present two patients diagnosed with *DSM*-5 Section II BPD, crossing between two therapists over the course of 3 years of psychodynamic psychotherapy. Treating clinicians rated patients for their respective treatment phases using the Level of Personality Functioning Scale (LPFS), capturing severity, and the Personality Inventory for the *DSM*-*5* (PID-5), capturing style. AMPD diagnostic profiles differentiated patients with BPD in both severity and style, and captured within-patient change beyond within-therapist response bias. Results indicated greater improvements in personality severity while personality style remained more stable. Implications for the patients’ treatment progress and associated challenges are discussed, as are considerations for the utility of the AMPD in therapy.

## Introduction

The Alternative Model for Personality Disorders (AMPD) of the *Diagnostic Statistical Manual of Mental Disorders* (5th edition; *DSM-5*; American Psychiatric Association, 2013) is a dimensional model of personality and personality pathology. The AMPD aimed to improve upon the well-documented limitations of the *DSM*’s categorical model of personality disorders (PDs), a model left unrevised since 1980 (Skodol et al., 2014; Bender et al., 2018; Waugh, 2019). AMPD personality conceptualization includes a personality (dys)function severity dimension (Criterion A) and maladaptive personality traits (Criterion B). Aligning with psychodynamic, interpersonal, and personological traditions (Waugh et al., 2017; Pincus and Roche, 2019), Criterion A reflects an underlying dimension of *personality pathology severity* defined by difficulties in self and interpersonal relatedness (Bender et al., 2011; Pincus et al., 2020). Through the Level of Personality Functioning Scale (LPFS; American Psychiatric Association, 2013), Criterion A represents the dynamic processes of perceiving, experiencing, and relating to the self (identity and self-direction) and others (empathy and intimacy). Criterion B aligns with the multivariate and empirical personality traditions and specifies interindividual differences in *maladaptive style*, or the characteristic, patterned expression of traits across contexts (Waugh et al., 2017; Krueger, 2019). The Personality Inventory for DSM-5 (PID-5; Krueger et al., 2012) operationalizes Criterion B as five dimensional trait domains and 25 dimensional trait facets. Taken together, the AMPD unites process and structure to offer a nuanced conceptualization of personality as severity of difficulties in self and relatedness that is further clarified by characteristic style.

### Clinical Utility of the Alternative Model for Personality Disorders

Since its inception, AMPD research has boomed with focus on the psychometric properties, validity, and reliability of Criterion A and Criterion B (e.g., Krueger et al., 2012; Few et al., 2013; Morey, 2017; Zimmermann et al., 2019; Bliton et al., 2021). However, exploring the clinical application and utility of the AMPD has received comparatively less attention. Clinical utility pertains to practical application of a clinical construct and is generally evaluated across three domains: (1) the communicative value among clinicians and patients, (2) practical implementation spanning accuracy, ease of use, and feasibility; and (3) usefulness in treatment planning and intervention (First et al., 2004; Reed, 2010; Mullins-Sweatt et al., 2016). Milinkovic and Tiliopoulos (2020) conducted a systematic review of the AMPD’s clinical utility among licensed and training clinicians and inpatient, outpatient, and forensic populations. Across 20 relevant studies, the results indicated (1) favorable communicative value between clinicians and between clinicians and patients, (2) high diagnostic accuracy and learnability of the model, and (3) helpful facilitation of appropriate intervention selection and clinic-decision making.

Clinical case examples have offered a unique perspective of the AMPD’s ability to translate from bench to bedside. Existing AMPD case examples provide illustrations of the AMPD’s clinical utility spanning case conceptualization, differential diagnosis, treatment planning, and intervention. Skodol et al. (2014) presented a comprehensive case example in which the AMPD profile (i.e., Criterion A and Criterion B assessment) distinguished personality pathology from depressed mood and informed interventions. Bach et al. (2015) contrasted six clinical cases to illustrate how AMPD profiles provide “individualized assessment,” clarify diagnoses, and refine case conceptualization (p. 19). Pincus et al. (2016) employed AMPD profiles to differentiate diagnostic profiles, case conceptualization, and specific interventions across three clinical cases of patients diagnosed with DSM-5 Section II narcissistic personality disorder. Specifically, Pincus et al. (2016) illustrated how initial therapeutic interventions, including the timing and delivery of diagnostic feedback, may differ across patients with varying levels of severity and trait constellations. Taken together, extant clinical case examples demonstrate the AMPD’s ability to differentiate diagnostic profiles within and across categorical personality disorder diagnoses and subsequently inform treatment planning in the initial phases of psychotherapy. However, less is known about the AMPD’s utility over the course of treatment. Thus, there is a need to examine changes in AMPD diagnostic profiles and personality expression within treatment and across time.

The AMPD profile illustrates *how* patients are impaired rather than simply *if* patients are impaired (Bach et al., 2015). Although they are complements, differences in Criterion A severity and Criterion B style have important implications for treatment planning and intervention implementation (see Hopwood, 2018 for a summary). Attunement to the patient’s overall severity of dysfunction informs broad clinical decision-making regarding the need for structure within session and boundaries across session, identification of patterns of relatedness, and flexibility in responding to acute distress, to name a few (Bateman, 2012; Bateman et al., 2015; Clarkin et al., 2015). The patient’s style directs how interventions are adapted and delivered to best meet the patient’s needs (McWilliams, 2011; Torres-Soto et al., 2018). Although relatively stable, personality traits can become more adaptive over time with intervention (Roberts et al., 2017). As such, a focal aim of treatment for personality style would be to reduce problematic expression rather than reconfigure trait constellation (Hopwood, 2018). Clinical case examples exemplifying how Criterion A and Criterion B guide interventions across sessions and subsequently engender personality change are warranted.

### Applying the Alternative Model for Personality Disorders to Borderline Personality Disorder Assessment and Intervention

Borderline personality pathology is a particularly compelling candidate to elucidate the AMPD’s clinical utility over time. The conceptualization of “borderline” is double-barreled. Borderline can refer to the heterogeneous, albeit discrete, borderline personality disorder (BPD). Stemming from psychoanalytic models (Kernberg, 1988), borderline can also be considered a spectrum of personality organization that undergirds all PDs and speaks to the dimensional severity of identity integration, maturity of defenses, and reality testing. Given the lack of clarity and contention surrounding BPD conceptualization (Gunderson et al., 2018), empirical efforts have focused on parsing apart the heterogeneity of BPD. Across analytic approaches, two robust overarching findings have emerged. First, modeling the factor structure of DSM criteria for BPD tend to support a single-factor solution rather than a multidimensional model (Hallquist and Pilkonis, 2012). Thus, BPD criteria are surmised to represent a common core of personality pathology with little variance remaining in a discrete borderline category after modeling a general personality pathology factor (Sharp et al., 2015; Wright et al., 2016). Second, the application of class or cluster analyses to DSM criteria or key symptom expressions of BPD (e.g., interpersonal difficulties and affective dysregulation) derive subgroups of individuals with BPD. Results have ranged from identifying subgroups differentiated by severity (Clifton and Pilkonis, 2007), internalizing versus externalizing subgroups (Smits et al., 2017; Johnson and Levy, 2020), and two to four classes of subgroups demarcated by core symptoms (e.g., Lenzenweger et al., 2008; Hallquist and Pilkonis, 2012; see Gamache et al., 2021 for a review). Taken together, efforts to understand BPD’s heterogeneity largely emphasize either severity or style.

As severity and style are both embedded within the AMPD, AMPD profiles offer a comprehensive framework to define borderline pathology. Gamache et al. (2021) found four AMPD profiles for BPD: (i) borderline traits, (ii) moderate pathology with impulsivity, (iii) moderate pathology with identity problems and depressivity, and (iv) severe pathology. The AMPD BPD profiles largely were distinguished by increasing severity level; however, results also pointed to the importance of Criterion A identity and Criterion B depressivity, impulsivity, and risk-taking in differentiating profiles. As such, the AMPD profile accounts for BPD as general personality pathology severity (i.e., genus) further explicated by characteristic symptom expression (i.e., species), a combination that seems necessary to understand BPD. Thus, investigating BPD through an AMPD lens as it directly applies to case conceptualization and treatment course offers importance insights into the AMPD’s clinical utility.

Individuals with BPD utilize significantly more treatment resources than individuals with mood, anxiety, or other personality disorders (Ansell et al., 2007). Borderline pathology accounts of approximately 10–20% of patients in outpatient settings (Korzekwa et al., 2008; Zimmerman et al., 2008); however, it remains difficult to follow patient treatment trajectory and associated symptom change over time. One major reason for this is discontinuity in treatment due to misdiagnosis and inconsistent case conceptualization, as it is not uncommon for individuals with BPD to only be properly diagnosed several years after first treatment contact (Biskin and Paris, 2012; Kjær et al., 2016). In a study comparing outpatients with BPD to those with other personality disorders and those with schizophrenia, 97% of the individuals with BPD reported a history of prior outpatient treatment compared to 33% of those with other personality disorders and 80% of those with schizophrenia (Skodol et al., 1983). Although numerous rounds of outpatient treatment certainly reflect the chronicity and longstanding nature of the difficulties those with BPD experience, disruptions in treatment continuity and changes in providers also frequently occur due to high rates of hospitalization, patient drop out, and therapist burnout (Zanarini et al., 2001; Wnuk et al., 2013). When patients shift in and out of therapy from one provider to another, the potential for assessment of patient change over time is often complicated or thwarted.

### Present Study

Following patient trajectories across therapists offers a unique opportunity to examine the AMPD’s sensitivity to and utility for capturing personality change over time for patients with BPD. As such, the present study aims to highlight the utility of the AMPD assessment framework for two clinical cases seen in a doctoral training clinic in three distinct ways: (i) highlighting heterogeneity in BPD between patients, (ii) comparing improvements in personality severity and style, and (iii) elucidating profile change across therapist ratings. To achieve these aims, we present two patients diagnosed with *DSM-5* Section II BPD who cross over between two therapists over the course of three years of psychodynamic psychotherapy. Patients’ diagnostic profiles were assessed with the AMPD framework for their respective time of treatment culminating in ratings across two phases of treatment. Aligning with the aims, we expect the AMPD diagnostic profile to differentiate between patients with BPD in both severity and style. Further, across therapist and time, we expect improvements in personality severity while personality style is predicted to remain more stable. Finally, we expect to see the AMPD profile capture within-patient change beyond within-therapist response bias through both treatment phases. As a set, the present aims will join extant efforts to demonstrate the AMPD’s clinical utility.

## Materials and Methods

### Measures

#### Level of Personality Functioning Scale

The Level of Personality Functioning Scale (LPFS; American Psychiatric Association, 2013) operationalizes Criterion A and rates the personality pathology severity. The LPFS includes the indicators of identity, self-direction, intimacy, and empathy. Impairment within the indicators is stratified across five distinct levels ranging from *Little to No Impairment* (Level 0) to *Extreme Impairment* (Level 4). Clinicians rated level of impairment across each indicator and derived total severity by the average of the indicators.

#### Personality Inventory of DSM-5 Informant Form

The Personality Inventory of DSM-5 – Informant Form (PID-5-IRF; American Psychiatric Association, 2013) operationalized Criterion B. The PID-5-IRF contained 220 items that derive five maladaptive trait domains and 25 maladaptive trait facets. Clinicians rated patients on each item ranging from (0) *Very False or Often False* to (3) *Very True or Often True*.

### Rating Procedure

Patients were first seen for an extensive psychodiagnostic assessment including psychosocial history, the Anxiety Disorders Interview Schedule for DSM-5 – Lifetime Version (ADIS; Di Nardo et al., 1994), and the International Personality Disorders Examination (IPDE; Loranger et al., 1994). Both patients met criteria for DSM-5 Section II BPD. Both patients were seen for psychodynamic psychotherapy over the course of three years provided by two doctoral-level training clinicians supervised by a licensed clinical psychologist specializing in personality disorder treatment and assessment. Both clinicians are white women, 29 and 30 years old, and presently in their 6th year of doctoral training.

**FIGURE 1:**
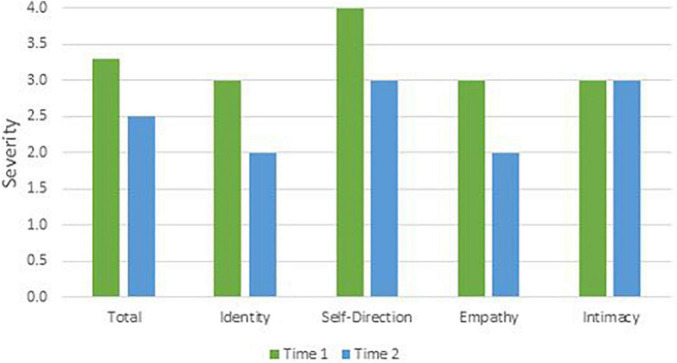
Mr. D’s change in LPFS severity.

**FIGURE 2:**
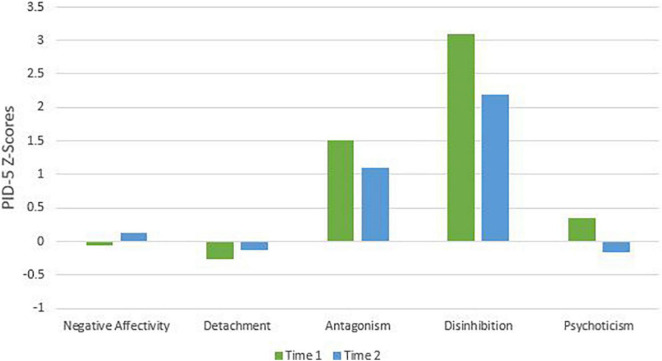
Mr. D’s change in PID-5 Z-scores by domain.

**FIGURE 3:**
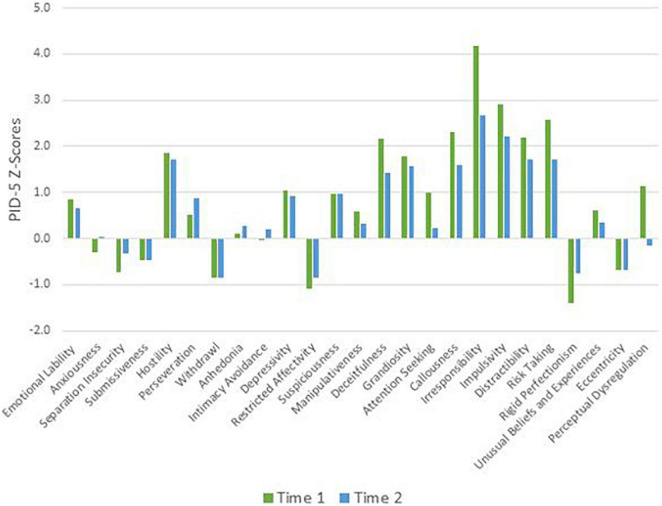
Mr. D’s change in PID-5 Z-scores by facet.

**FIGURE 4:**
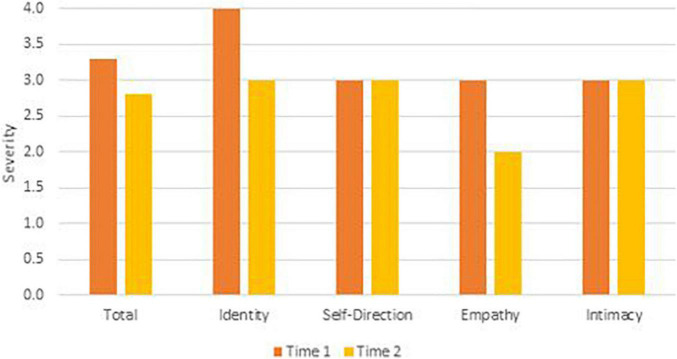
Ms. B’s change in LPFS severity.

**FIGURE 5:**
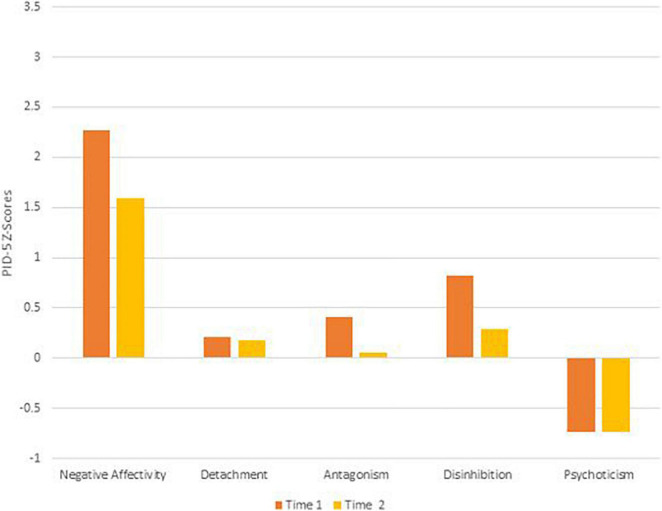
Ms. B’s change in PID-5 Z-scores by domain.

**FIGURE 6:**
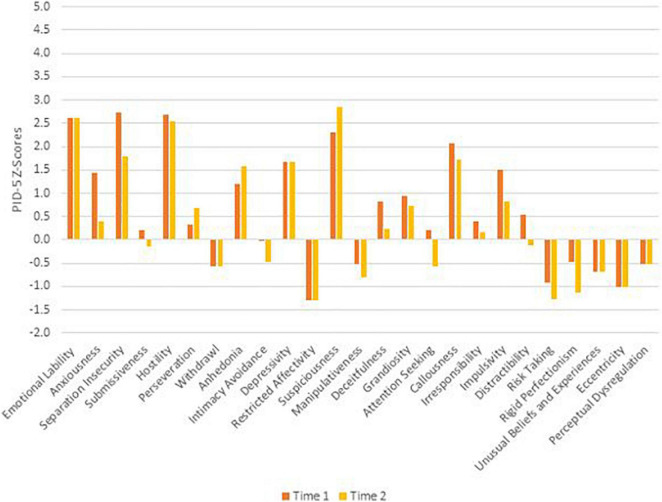
Ms. B’s change in PID-5 Z-scores by facet.

To remain faithful to the AMPD, patients’ AMPD diagnostic profiles are assessed using the Level of Personality Functioning Scale (LPFS) and the Personality Inventory for the DSM-5 – Informant Form (PID-5-IRF) as specified within Section III of the *DSM-5*. Each of the two treating clinicians retrospectively rated the patients as they presented during the first 2 months of their respective phases of treatment, culminating in ratings across two phases of treatment. Ratings were discussed with the supervising clinician and each therapist was blind to the other’s ratings. LPFS ratings are presented in Table 1 and Figure 1 for Mr. D, and Figure 4 for Ms. B. PID-5 ratings were standardized using informant norms (Markon et al., 2013) and are presented in Figures 2, 3 for Mr. D and Figures 5, 6 for Ms. B. Care was taken to alter identifying information and case material to protect the confidentiality of the patients.

**TABLE 1:** Clinician-rated level of personality functioning over time.

*Personality functioning*	Mr. D		Ms. B	
	*Time 1*	*Time 2*	*Time 1*	*Time 2*
Self				
Identity	3	2	4	3
Self-direction	4	3	3	3
Interpersonal				
Empathy	3	2	3	2
Intimacy	3	3	3	3
Total	3.3	2.5	3.3	2.8

## Results

### Mr. D

Mr. D is a White man between the ages of 25 and 30 years old with a history of impulsive gambling, substance abuse, and difficulties maintaining employment. Following numerous hospitalizations and substance related legal involvement, Mr. D was referred for individual psychotherapy. He presented with concerns related to affective lability, substantial impulsivity, dysfunctional relationships, suicidal ideation, and difficulty following through on goal-oriented behaviors. Upon diagnostic assessment, it became apparent that Mr. D’s symptoms and experiences could be best understood through the lens of personality pathology. He received a primary diagnosis of borderline personality disorder with antisocial traits. Mr. D also met criteria for a severe substance use disorder and recurrent, moderate episodes of major depression. He began weekly individual transference focused psychotherapy (TFP; Caligor et al., 2018), increasing to twice weekly sessions after several months, with additional medication management and Dialectical Behavior Therapy skills group. Mr. D was transferred from the first to second therapist after 2.5 years and has been working with the second for approximately 6 months.

#### Time 1

At Time 1, Mr. D’s LPFS score showed severe impairment in personality functioning (Table 1). Although the LPFS model is understood as a unidimensional index of severity (Bliton et al., 2021), nuanced clinical information regarding personality functioning can be gleaned from the four domains of identity, self-direction, intimacy, and empathy. Depicted in Figure 1 at Time 1 (green bars), Mr. D showed extreme impairment in self-direction and severe impairment in identity, intimacy, and empathy. His PID-5 domain elevations were best characterized as disinhibition (>3 SDs above the mean) and antagonism (>1 SD above the mean; Figure 2). As illustrated in Figure 3, PID-5 facet elevations included irresponsibility (>4 SDs above the mean), impulsivity, risk-taking, distractibility, callousness, deceitfulness (>2 SDs above the mean), hostility, grandiosity, perceptual dysregulation, and depressivity (>1 SD above the mean). Per the AMPD, an individual can meet criteria for the legacy BPD category if each of the following proposed diagnostic criteria are met: (a) moderate or greater impairment in personality functioning within at least two of the four LPFS domains of identity, self-direction, empathy, or intimacy and (b) elevations on four or more of the following maladaptive traits: emotional lability, anxiousness, separation insecurity, depressivity, impulsivity, risk-taking, or hostility. Notably, one of the four traits must be either impulsivity, risk-taking, or hostility (American Psychiatric Association, 2013). Mr. D’s AMPD profile meets criteria for the BPD legacy category as all four LPFS domains at a moderate or greater level of severity and four of the listed maladaptive traits are elevated—depressivity, impulsivity, risk-taking, and hostility.

#### Time 2

At Time 2, Mr. D’s LPFS score illustrated moderate to severe impairment in personality functioning (Table 1). Depicted in Figure 1 at Time 2 (blue bars), Mr. D evidenced severe impairment in self-direction and intimacy and moderate impairment in identity and empathy. His PID-5 domain elevations remained characterized by disinhibition (>2 SDs above the mean) and antagonism (>1 SD above the mean; Figure 2). Further, his PID-5 facet level elevations included irresponsibility and impulsivity (>2 SDs above the mean) as well risk-taking, hostility, distractibility, callousness, grandiosity, and deceitfulness (>1 SD above the mean). Of note, Mr. D’s AMPD profile at Time 2 no longer meets criteria for the BPD legacy category. Although he continued to show moderate to severe impairment across LPFS domains and elevation in impulsivity, risk-taking, and hostility, his trait profile showed reduction in depressivity (<1 SD above the mean), leaving only three out of four required traits elevated within the BPD legacy criteria.

### Ms. B

Ms. B is a White woman between the ages of 55 and 60 who was underemployed relative to her education and training. Ms. B presented for services after a former provider recommended a thorough diagnostic assessment for suspected personality pathology. At the diagnostic assessment, Ms. B’s presenting concerns included a long history of treatment-resistant depression and anxiety related to relationship difficulties, specifically an estranged relationship with her daughter, employment difficulties, and a generally unfulfilling life. Ms. B’s diagnostic assessment affirmed BPD as the principal diagnosis with dependent and narcissistic traits. Further, Ms. B was also diagnosed with generalized anxiety disorder and recurrent, moderate major depressive episodes. Prior to Time 1, Ms. B engaged in TFP and participated in a Dialectical Behavior Therapy skills group for roughly a year. Additionally, Ms. B benefited from local case management services. At Time 1, Ms. B’s personality pathology had been clarified and adjusted to reflect comorbid borderline and dependent personality disorders, and the generalized anxiety disorder diagnoses was retained. Ms. B has continued to engage in individual TFP, a skills group, and case management services. Ms. B was transferred from her first to second therapist after 1.5 years and has been working with the second therapist for another 1.5 years.

#### Time 1

At Time 1, Ms. B’s LPFS score illustrated severe impairment in personality functioning (Table 1). As illustrated in Figure 4 at Time 1 (orange bars), Ms. B exhibited extreme impairment in identity and severe impairment in self-direction, empathy, and intimacy. As illustrated in Figure 5, her PID-5 domain elevation was best characterized as negative affectivity (>2 SDs above the mean). Shown in Figure 6, her PID-5 facet level elevations included separation anxiety, hostility, emotional lability, suspiciousness, and callousness (>2 SDs above the mean) as well as depressivity, impulsivity, anxiousness, and anhedonia (>1 SD above the mean). Per the previously mentioned criteria, Ms. B’s Time 1 AMPD profile also meets criteria for the BPD legacy category with all four LPFS domains at a moderate or greater level of severity and at least four elevated maladaptive traits including emotional lability, anxiousness, separation insecurity, depressivity, impulsivity, and hostility.

#### Time 2

At Time 2 Ms. B’s LPFS score indicated moderate to severe levels of impairment in personality functioning (Table 1). Looking at domains of the LPFS shown in Figure 4 at Time 2 (yellow bars), Ms. B continued to show severe impairment in self-direction, intimacy, and identity, and moderate impairment in empathy. As illustrated in Figure 5, her PID-5 domain elevations were best characterized by negative affectivity. Examining facet scores across the PID-5 domains in Figure 6, Ms. B showed elevations in emotional lability, hostility, and suspiciousness (>2 SDs above the mean) as well as separation insecurity, anhedonia, depressivity, and callousness (>1 SDs above the mean). Although she showed a reduction in anxiousness and impulsivity (<1 SD above the mean), Ms. B’s AMPD profile continued to meet criteria for the legacy BPD category at Time 2 with all four LPFS domains within the moderate to severe level of impairment and continued elevation in four maladaptive traits including emotional lability, separation insecurity, depressivity, and hostility.

### Profile Associations

To ensure patients’ AMPD profile changes were not an artifact of within-clinician rating bias and to examine the stability of AMPD profiles across time and therapist, we compared the associations between AMPD profiles within patients and within therapists. Patients’ AMPD profiles (i.e., four LPFS indicators and 25 PID-5 facets) at Time 1 and at Time 2 were more strongly associated (*r_*Mr. D*_* = 0.96, *p* < 0.001; *r_*Ms. B*_* = 0.96, *p* < 0.001) than within-clinician ratings (*r_*CFB*_* = 0.60, *p* < 0.001; *r_*LKR*_* = 0.48, *p* < 0.01).

## Discussion

The current presentation followed patient trajectories across therapists to examine the AMPD’s sensitivity to and utility for capturing personality stability and change over time for two patients with BPD. We specifically sought to highlight the utility of the AMPD assessment framework for two clinical cases seen in a doctoral training clinic in three distinct ways: (i) highlighting heterogeneity in BPD between patients, (ii) comparing improvements in personality severity and style, and (iii) elucidating profile change across therapist ratings.

### Mr. D’s Alternative Model for Personality Disorders Profile Over Time

At Time 1 during the initial phase of treatment, Mr. D’s AMPD profile was defined by severe impairment in personality functioning which indicates significant difficulties in self and relational functioning. Aligning with psychodynamic theoretical origins, the indicators of identity, self-direction, empathy, and intimacy do not exist as discrete factors but instead as interconnected processes. As such, Mr. D suffered from an incoherent and unstable identity. His sense of self largely vacillating between an idealized care-free, excitable self and a deeply loathed incapable and flawed self. This unstable and unintegrated sense of self largely impacted Mr. D’s capacity for self-directedness. He struggled to mentalize his own behavior particularly when it came to treatment engagement and occupational functioning. Mr. D floated from one job to the next, either getting fired for poor performance or quitting prior to being let go. He had difficulty reflecting on his own behavior, tending to spiral into self-doubt and deep levels of shame when beginning to acknowledge the impact his behavior had on others and would often defend against this by completely splitting off taking another’s perspective. Mr. D often exhibited the idealized self via irresponsibility and recklessness, while associating being responsible with the risk of exposing incompetence and being shamed. Although Mr. D had several people in his life with whom he socialized, he lacked intimacy in close relationships where a similar conflict between wanting closeness and fearing being fully seen contributed greatly to his difficulties experiencing himself and others as consistent, and he frequently pushed others away.

These core impairments in self and relational functioning presented in conjunction with an impulsive, disorganized, and at times antagonistic personality style. Mr. D repeatedly tested the treatment frame, missing session entirely or arriving late (impulsivity, irresponsibility). The same pattern was reported to occur within the work setting and Mr. D often struggled to take responsibility for this, becoming hostile and dismissive when his own responsibility for the treatment was confronted (hostility). This was further exemplified by continued legal involvement secondary to substance related charges (irresponsibility; deceitfulness). Early treatment goals largely centered on containing acting out behaviors such as substance use, risky sexual behavior, and inconsistent attendance, increasing Mr. D’s capacity to integrate a sense of responsibility without risk of shame, helping Mr. D to tolerate imperfection in intimate relationships, and to set reasonable proximal and distal goals for himself in terms of employment.

At Time 2, Mr. D’s AMPD profile exhibited moderate to severe impairment in personality functioning. Mr. D’s identity still tended to vacillate between an idealized, care-free self and an incapable and flawed self; however, Mr. D demonstrated an improved capacity to reflect on this vacillation in therapy. Further, Mr. D was beginning to recognize this pattern of devaluing in order to maintain his idealized self-image and, thus, protect against the risk of shame. Mr. D was beginning to hold, although sometimes briefly, a more integrated view of his identity. Aligning with identity consolidation, Mr. D’s goal-directed functioning had improved as he was consistently maintaining employment. However, he continued to carelessly approach responsibilities such as arriving late to session, struggling with timeliness and task completion at work, and neglecting communal chores within his home environment. Mr. D demonstrated a tenuous though growing recognition of the consequences and impact of his behavior; however, this insight amplified his experience of shame and guilt. Mr. D still struggled to hold that others may have thoughts, motivations, and reactions that were different from his own, and he lacked close relationships built on mutuality and transparency.

Despite improvements in personality functioning severity, the structure of Mr. D’s personality style remained consistent, even with a reduction in the intensity of maladaptive expression. Despite some improvements, Mr. D still had difficulty honoring interpersonal and professional obligations and commitments (irresponsibility) which further complicated important relationships and goal-directed behavior. Mr. D’s irresponsibility continued to interact with his tendency to immediately seek momentary gratifications without considering impact (impulsivity). As such, it was common for Mr. D to find himself in difficult situations for which he needed to take responsibility. Mr. D would subsequently externalizing blame to others in efforts to defend against shame, a process that was often explored within the therapy room. Thus, Mr. D’s treatment goals remained geared toward an increased capacity to integrate a sense of responsibility without shame, tolerance of mutual intimacy in relationships, and setting achievable professional goals. Interventions focused on observing Mr. D’s responsibility, or lack thereof, across circumstances, and accurate recognition of Mr. D’s responsibility as it relates to his self-concept, career pursuits, and relationships.

### Ms. B’s Alternative Model for Personality Disorders Profile Over Time

Ms. B’s Time 1 AMPD profile was defined by severe impairment in all LPFS domains of personality functioning which speaks to great impediments in relating to self and relating to others. Ms. B suffered from an impoverished and unstable identity which depended on overt validation and caretaking from others for cohesion. However, Ms. B easily perceived persecution, criticism, and judgment from others and often felt alone, excluded, and abandoned. Taken together, Ms. B generally experienced herself as denied and rejected by a withholding, critical other. Ms. B had difficultly observing and understanding her inner world as she experienced contradictory internal standards for behavior which clouded her ability to observe the impact of her own oscillating aggressive and overly dependent behavior on others. Ms. B was caught in a vicious cycle of heavily depending on others for regulation, experiencing others as withholding and rejecting, and not recognizing that her own aggressive and dependent responses drove away and burned out others. Ms. B had experienced numerous relationships ruptures with important others leaving her with few relationships on which she could truly depend on. This lack of interpersonal support further confirmed Ms. B’s sense of herself as denied and rejected.

Ms. B’s central impediments in self and other functioning were further complicated by her personality style. Aligning with her tendency to experience herself as denied by a withholding other, Ms. B appeared stuck between expectations of interpersonal harm (suspiciousness) and a pervasive fear of rejection amplified by an impaired capacity to care for herself (separation anxiety). Thus, Ms. B seemed to desperately approach others for self-definition, regulation, and care despite the intense fear of harm. Ms. B appeared to have some insight into the intensity of her unstable emotions (emotional lability) though she greatly struggled with affect regulation. Ms. B’s negative affectivity fueled behavior toward others that vacillated from infantile dependence to indignant hostility. Given that Ms. B had limited awareness into her interpersonal impact, it was difficult for her to see beyond her own needs and observe the damaging impact her emotional cascades had on others. Notably, beyond Ms. B’s difficulty recognizing her antagonism and hostility on her own, when the therapist would bring it to her attention, Ms. B was largely unconcerned about others (callousness).

The beginning of treatment was marked by frequent intersession phone calls, requests for immediate sessions, and resistance to ending regularly scheduled sessions on time. This dependency held true even as Ms. B frequently experienced the therapist as withholding and rejecting. Ms. B would evade bids to reflect on her thoughts, emotions, and behavior and would instead revert to detailing past experiences that she felt justified her current experience and emotions. Ms. B’s aggression was wholly compartmentalized, and she would deny hostility while loudly interrupting and speaking over the therapist. To help Ms. B achieve her self-generated goals of feeling happier and developing more meaningful relationships, early interventions focused on maintaining a consistent treatment frame and helping Ms. B notice the vacillation between overly dependent and intensely aggressive behavior.

Ms. B’s Time 2 AMPD profile shifted to show moderate to severe levels of personality functioning. Her identity continued to exhibit a pattern of perceiving others as withholding and rejecting and therefore experiencing herself as deprived and abandoned. Ms. B had difficulty observing this process, showing a capacity to briefly reflect on this dynamic but quickly vacillating when this became threatening to her. Notably, Ms. B showed improvement in empathy regarding her ability to observe the impact of her aggression and dependency on others. Although intimacy and empathy remained impaired as she persisted in relying on other relationships for regulation, her capacity to hold and reflect on this within therapy increased. Ms. B demonstrated growing willingness to consider others’ perspectives, catching her aggression in the moment and taking a step back to consider how the other person was feeling. Although Ms. B largely continued to view others as sources of emotional regulation and feared rejection, her ability to observe this process began to emerge, allowing Ms. B to attempt to repair relationships following ruptures.

Despite improvement in overall level of personality functioning, Ms. B’s personality style remained consistent, albeit with a reduction in intensity of expression. Aligning with her tendency to experience herself as deprived by a withholding other and incapable of caring for herself (separation anxiety), Ms. B continued to experience expectations of interpersonal harm (suspiciousness). Thus, Ms. B continued to seek out others for self-definition, regulation, and care to a somewhat lesser degree as her insight into this process and general levels of emotional instability (emotional lability) increased as previously noted. When Ms. B’s needs for self-regulation or her expectations for care were not met, she continued to engage in aggressive behavior such as yelling and name calling (hostility). Although she demonstrated minimal concern for others when feeling deprived or rejected (callousness), Ms. B’s improving capacity for perspective taking and empathy allowed her to reflect on this in the moment. Overall, treatment goals continued to focus on helping Ms. B to acknowledge and observe her tendency to vacillate between dependency and hostility so that she could build and maintain the types of relationships and closeness that she desired. Specifically, bids to mentalize and perspective take were utilized frequently in session.

### Borderline Personality Disorder as a Heterogeneous Diagnosis

At Time 1, both Mr. D and Ms. B met criteria for the AMPD legacy BPD diagnosis. This is consistent with their diagnoses per the categorical section II DSM-5 criteria that were determined upon intake when presenting for treatment. These results are consistent with empirical studies that have found that given the substantial construct overlap, the traditional categorical BPD diagnosis can be reliably assessed by the AMPD (Mulay et al., 2019).

However, each patient presented with differing manifestations of this pathology, capturing the heterogeneity of BPD. Assessing the heterogeneity of BPD using the AMPD, we found that although both patients demonstrated severe levels of personality impairment per the LPFS, Ms. B’s most significant impairment fell within the domain of identity diffusion and Mr. D’s within the domain of self-direction. Looking at trait constellations per the PID-5, Ms. B showed elevation in emotional lability, separation insecurity, anxiousness, hostility, impulsivity, and depressivity. In comparison, Mr. D showed elevation in impulsivity, risk-taking, hostility, and depressivity. Although there is some overlap in traits between these two patients, Mr. D’s trait profile was most elevated in the domain of disinhibition while Ms. B’s was most elevated in the domain of negative affectivity. Interestingly, these differences in traits are consistent with Mr. D’s antisocial personality traits captured at intake evidenced here by impulsivity, recklessness, hostility, and risk-taking and Ms. B’s dependent personality traits evidenced by her high levels of separation insecurity and reliance on others for emotion regulation.

These differences in AMPD profiles, although yielding a similar diagnostic picture at first glance (i.e., BPD), mirror the patients’ stark differences in presentation that were seen clinically in session. Ms. B’s diffuse sense of identity and poor self-esteem primarily presented as extreme vacillations in affect and intense fears of abandonment leading first to hostility and anger and then urgent dependency when these needs were not met. Mr. D presented with high levels of impulsivity and risk-taking that resulted from a significant lack of coherence in his sense of self and self-direction which also tested the treatment frame. Given the abundance of research showing BPD to be a heterogenous diagnosis, it is not surprising that this also meets criteria for this legacy diagnosis. A recent study conducted by Gamache et al. (2021) sought to identify subgroups of patients meeting the legacy BPD category. Using latent Profile Analysis (LPA) of Criterion A and B facets, they found four distinct profiles: (1) borderline traits (characterized by relatively lower severity albeit impairment in self-direction and empathy as core traits of hostility, impulsivity, and risk-taking), (2) moderative personality severity with impulsivity and risk-taking (characterized by depressivity and increased impulsivity as well as slightly elevated impairment in identity functioning), (3) moderate personality severity with identity problems and depressivity (characterized by increased depressivity and moderate impairment in identity functioning), and (4) severe personality pathology (characterized by severe impairment in self-direction and empathy as well as elevation in hostility and impulsivity). Notably, Criterion A identity impairment and Criterion B traits of impulsivity and risk-taking emerged as key differentiating variables distinguishing profiles. Although at Time 1 neither Mr. D nor Ms. B seemed to fit these profiles exactly, both patients were rated as exhibiting severe identity impairment, particularly Ms. B, and struggling with impulsivity and risk-taking, particularly Mr. D. As such, the ratings seen here within a clinical setting appear to be in line with research on the translation of heterogeneous presentations of BPD to the AMPD framework.

### Clinical Change Captured by Criterion A and Criterion B

Aim 2 of this presentation focused on assessing changes in AMPD Criterion A and Criterion B across treatment periods with two consecutive therapists. Although both Ms. B and Mr. D met criteria for the legacy BPD category at Time 1, only Ms. B did so at Time 2. As illustrated in Figures 2, 3, 5, 6, there was not a reconfiguration of Criterion B trait domains and facets from Time 1 to Time 2 but rather a reduction in the maladaptive manifestations for both patients. This is consistent with literature on the general stability of personality traits over time (Ferguson, 2010). For example, at Time 1 Mr. D had elevated levels of irresponsibility (>4 SDs above the mean) and at Time 2 the irresponsibility facet remained elevated but had reduced (>2 SDs above the mean). Similarly, Ms. B showed elevated levels of separation insecurity (>2 SDs above the mean) at Time 1 that remained elevated but reduced at Time 2 (>1 SDs above the mean). However, despite maintaining general consistency in their trait profiles, both patients demonstrated a reduction in overall severity personality dysfunction over the course of treatment from Time 1 to Time 2 (Table 1 and Figures 1, 4). Both Ms. B and Mr. D were being treated from a psychodynamic orientation with transference focused psychotherapy (TFP). TFP is an empirically supported treatment for BPD organized around the theory that core deficits in BPD stem from the individual’s incoherent and split mental representations of self and other. This incoherence leaves the individual struggling to regulate emotions and behaviors in an adaptive manner and TFP aims to increase a patient’s capacity for accurate and coherent reflection on self and other (Clarkin et al., 2006; Caligor et al., 2018). These core deficits are central to Criterion A and previous research has suggested that BPD can be thought of as a general factor of personality disorder, loading almost exclusively onto measures of general severity (Sharp et al., 2015; Wright et al., 2016). Therefore, as noted by Hopwood (2018), it follows that a treatment targeting BPD would yield changes to Criterion A of the AMPD.

### Implications for Profile Change Across Therapist Ratings

As demonstrated by high within-patient profile correlations relative to within-therapist profile correlations, the AMPD appears to be sensitive to clinical change, particularly for personality functioning severity as reflected in Criterion A. Although Mr. D and Ms. B struggled with empathy, particularly at Time 1, Mr. D and Ms. B each pulled for unique countertransferential reactions that were similarly experienced by both therapists. Mr. D’s difficulty observing and tolerating the impact of his behavior on others, tended to leave both therapists feeling parentified in moments of his irresponsibility and rebelliousness. However, with Ms. B., her inability to see beyond the immediacy of her own affect and needs left both therapists feeling dismissed and ineffective when clinician responses to her repeated bids for help were promptly rejected. Although both patients struggled to understand the perspective of others, these difficulties manifested in different ways that are captured by Criterion B of the AMPD: Mr. D presenting as impulsive and Ms. B as affectively dysregulated. These differences between patients that were jointly experienced by each of the treating clinicians was mirrored in the LPFS and PID-5 ratings suggesting that the ratings reflect clinical phenomenon rather than rater bias.

As reflected in Criterion A improvements, Mr. D and Ms. B both grew in their capacity to mentalize. They better understood their own motivations and common mental states, and perspective-taking allowed for greater empathy. Even still, Mr. D’s trait level impulsivity and Ms. B’s trait level negative affectivity continued to be unique challenges, albeit with lesser intensity and disruption to functioning. Overall, this speaks to the changes observed in self in relation to other functioning and the relative stability of trait level differences. Although Criterion A and B provide a clear picture of progress as noted above, the AMPD also highlighted emerging targets for treatment. For example, as Mr. D gained insight into his difficulties in self and relatedness, he began to better understand the protective nature of his tendency to devalue in an effort to maintain an idealized self-image and protect against the risk of shame. Targeting his deeply rooted and critical self-concept became a new therapy goal. As Ms. B demonstrated a growing willingness to consider other’s perspectives, catching her aggression more readily in the moment, she began to see her impact on others. As such, working to more effectively repair relationships following ruptures became a focus of treatment.

### Conclusions, Limitations, and Future Directions

We aimed to demonstrate the clinical utility of the AMPD model across therapists and phases of treatment. Prior literature has defined clinical utility of a model by three main features: (1) communicative value; (2) implementation characteristics; and (3) usefulness in selecting proper interventions and making clinical decisions (Reed, 2010; Mullins-Sweatt et al., 2016). The current case reports demonstrate that assessment of the AMPD, particularly for patients cycling through numerous therapists over time, appears to be fruitful. In addition to providing a useful framework for understanding improvement, easy implementation of the LPFS and PID-5 on the part of the clinician allows for a rich clinical profile and description to be created with little time or cost. Knowledge of a patient’s severity of personality impairments and the specific style in which they manifest aids the new clinician beginning work with these challenging patients by providing useful information to guide treatment planning.

The current study has a number of limitations. First, therapists made retrospective ratings on the LPFS and PID-5 based on the patients’ presentations in the early months of each treatment phase. Ideally such ratings would be made at an optimal point following initiation of treatment with each therapist. Both patients were still in treatment with their second therapists at the time of the ratings and both therapists and the clinical supervisor had significant experience with the cases over the entire 3 years of treatment. Although retrospective ratings are a limitation, the pattern of profile associations within patients relative to therapists supports the validity of the ratings. Second, this article focused exclusively on BPD and psychodynamic psychotherapy. Future exploration of the AMPD’s clinical utility in assessing the full spectrum of personality functioning (i.e., non-pathological to pathological) and in conjunction with other diagnostic presentations is warranted. A unique facet of the present study was the transfer between two clinicians allowing for patient assessment over time and across raters within a naturalistic clinical setting. In keeping with prior literature on the AMPD (e.g., Milinkovic and Tiliopoulos, 2020), we recommend assessment using the AMPD be investigated within the context of numerous treatment orientations such as TFP, Dialectical Behavior Therapy, and Schema-focused Therapy, where the goals of improving personality functioning are central.

Overall, it appears the AMPD can account for distinct presentations of BPD pathology, greatly improving upon the information provided with a categorical diagnosis. Although both patients were diagnosed with BPD at intake assessment, the AMPD framework shed light on each patient’s distinct core deficits in functioning and style of presentation that were experienced in therapy. Mr. D presented with significant challenges in areas of irresponsibility, risk-taking, and externalizing and greatly feared exposing incompetence and flaws whereas Ms. B presented with affective dysregulation, anxiety, hostility, and an overall internalizing style and showed high levels of dependency and fears being deprived and abandoned. In conjunction with the ease of use of the LPFS and PID-5 rating scales, the present study highlights the benefits of the AMPD in clinical practice, particularly for better understanding borderline pathology and following personality change over time.

## Data Availability Statement

The raw data supporting the conclusions of this article will be made available by the authors, without undue reservation.

## Author Contributions

CB, LR, and AP contributed to the design of the work. CB and LR rated and analyzed the data, and contributed to the interpretation of the data and drafting of the work. AP provided oversight for the data rating. All authors contributed to the article and approved the submitted version.

## Conflict of Interest

The authors declare that the research was conducted in the absence of any commercial or financial relationships that could be construed as a potential conflict of interest.

## Publisher’s Note

All claims expressed in this article are solely those of the authors and do not necessarily represent those of their affiliated organizations, or those of the publisher, the editors and the reviewers. Any product that may be evaluated in this article, or claim that may be made by its manufacturer, is not guaranteed or endorsed by the publisher.
